# Implications of the “Language as Situated” View for Written Iconicity

**DOI:** 10.5334/joc.159

**Published:** 2021-08-23

**Authors:** David M. Sidhu, Penny M. Pexman

**Affiliations:** 1 University College London, GB; 2 University of Calgary, CA

**Keywords:** Visual word processing, Auditory word processing, Language production

## Abstract

In their review, Murgiano, Motamedi, and Vigliocco (2020) lay out a new perspective in which they argue that language should be understood as a situated phenomenon. This perspective has implications for the study of written language, which is fundamentally an un-situated phenomenon. We consider the implications of this perspective for iconicity as it appears in written language. We argue that typical methods for studying word processing (e.g., the lexical decision task) may be bound to underestimate the relevance of iconicity for language. In addition, the typical approach of collecting ratings of individual words on a lexical-semantic dimension may not be well suited to quantifying iconicity. Nevertheless, we believe the field should continue to explore effects of iconicity in language processing, and we discuss some potential ways to adjust traditional word processing tasks.

We are in agreement with Murgiano et al. (2020) that non-arbitrariness in language is “more than marginal”, and that iconicity is fundamentally a situated phenomenon. We think that this perspective has important implications for research that considers the role of iconicity in the processing of written language. By its nature written language is an un-situated phenomenon; it does not provide cues such as prosody, gesture, or points which often accompany spoken language. Murgiano et al. describe one way in which iconicity can exist in purely written form, when they discuss iconicity as it exists in “language as a system”: words whose forms have iconic links with their meaning (*lexicalized iconicity*; e.g., onomatopoeia). Yet even this form of iconicity may be impoverished when it appears in writing as opposed to face-to-face communication. We believe this view has important implications for how iconicity is studied. In particular, it is possible that traditional lexical tasks are bound to underestimate iconicity’s role in language.

A mainstay of word recognition research is the lexical decision task (*LDT*): on each trial, participants are shown a letter string and asked to decide if it is a real word or not. Various lexical and semantic features of words have been shown to affect how quickly those words can be processed on a lexical decision task. Indeed, as reviewed by Murgiano et al. (2020), iconicity has been shown to be one such factor, with iconic words (in particular onomatopoeia) processed faster than arbitrary words (Meteyard et al., 2015; Sidhu et al., 2020). However, if iconicity is fundamentally a situated phenomenon, such tasks may underestimate effects of iconicity. It may be that, for instance, a word must be pronounced aloud, perhaps even with prosodic embellishment, for its iconicity to truly emerge (see Dingemanse et al., 2016). For instance, reading “the wind made a whooshing sound” (already more situated that just seeing “whoosh” presented in LDT) is quite different from hearing this sentence said aloud, while drawing out the /ʃ/ sound. Furthermore, there is evidence that the LDT directs participants towards the *visual* features of a referent (see Lynott et al., 2020), which tend not to be iconically related to a word’s form (Winter et al., 2017). Thus, it is perhaps not surprising that while we found effects of iconicity in one set of LDT experiments (Sidhu et al., 2020), we also failed to observe such effects in another (Cnudde et al., in prep). We have, further, failed to observe effects of iconicity in a semantic decision task (i.e., “Is this object large or small?”; Sidhu & Pexman, in review). This variability is consistent with the inference that effects of written iconicity are ephemeral, likely to vary across different lists and tasks. In addition, existing models of word recognition (e.g., Coltheart et al., 2001; Harm & Seidenberg, 2004) have a difficult time incorporating situated phenomena like iconicity (for some discussion see Sidhu et al., 2020). This is because such models imagine static properties and processes.

The traditional method of measuring a dimension in lexical semantic research is to ask participants to rate words on a Likert scale. Iconicity ratings (Perry et al., 2015; Winter et al., 2017) have been collected in this fashion and have been extremely useful, enabling a variety of studies by providing a way of quantifying iconicity (e.g., Dingemanse & Thompson, 2020; Sidhu et al., 2020). We wholeheartedly endorse their continued use as it is clear that these ratings *do* tap into iconicity. They have high face validity: words that should have a high iconicity rating, such as onomatopoeia, appear at the top of the rating scale. These iconicity ratings also correlate with dimensions that one would expect to correlate with iconicity, such as semantic neighbourhood density (Sidhu & Pexman, 2018) or sensory experience ratings (Winter et al., 2018). Nevertheless, it is worth considering the extent to which there are aspects of a word’s iconicity that only emerge in context, and when presented with other cues (e.g., prosody; see Dingemanse et al., 2016). Indeed, there is evidence for contextual variability in iconicity ratings, as Perry et al. (2015) found only a moderate correlation (*r* = .61) between iconicity ratings collected with written vs. auditory presentation.

What might be the relationship of these subjective iconicity ratings of an isolated word, to a word’s “true” iconicity? Perhaps they represent the *baseline* of a word’s iconicity, which can only be increased when it appears in context. Another possibility is that they represent a word’s *potential* for iconicity, which can then either be capitalized on or not, and to different degrees, when used in context. With this in mind a word’s iconicity becomes more of a distribution of potential realizations than a single value.

We do believe that the field should continue to explore the role of iconicity in written language, but with the caveat that the behaviour of lexicalized iconicity on impoverished lexical processing tasks may be an underestimation. There may be ways to adapt traditional tasks used in cognitive psychology to increase the amount of iconicity that can be captured. For one, there is already evidence that auditory presentation leads to greater iconicity effects in word recognition than does visual presentation (Meteyard et al., 2015). It could be worthwhile to explore auditory presentations with prosodic intonation, and to consider presenting whole sentences rather than isolated words, or even video presentations. It could also be worthwhile to explore other methods of collecting iconicity ratings, perhaps by presenting words in context. One might also explore complementary ways of deriving iconicity besides Likert scale ratings (for a recent attempt see Sidhu et al., 2021). On a larger scale, the issues that Murgiano et al. (2020) raise regarding the situatedness of language bring to light important questions and future challenges for lexical processing research.
